# Single-energy CT predicts uric acid stones with accuracy comparable to dual-energy CT—prospective validation of a quantitative method

**DOI:** 10.1007/s00330-021-07713-3

**Published:** 2021-02-26

**Authors:** Johan Jendeberg, Per Thunberg, Marcin Popiolek, Mats Lidén

**Affiliations:** 1 Department of Radiology, Faculty of Medicine and Health, Örebro University, 70185 Örebro, Sweden; 2 Department of Medical Physics, Faculty of Medicine and Health, Örebro University, Örebro, Sweden; 3 Department of Urology, Örebro University Hospital, Örebro, Sweden

**Keywords:** Urolithiasis, Uric acid, Urinary calculi, Multidetector computed tomography

## Abstract

**Objectives:**

To prospectively validate three quantitative single-energy CT (SE-CT) methods for classifying uric acid (UA) and non-uric acid (non-UA) stones.

**Methods:**

Between September 2018 and September 2019, 116 study participants were prospectively included in the study if they had at least one 3–20-mm urinary stone on an initial urinary tract SE-CT scan. An additional dual-energy CT (DE-CT) scan was performed, limited to the stone of interest. Additionally, to include a sufficient number of UA stones, eight participants with confirmed UA stone on DE-CT were retrospectively included. The SE-CT stone features used in the prediction models were (1) maximum attenuation (maxHU) and (2) the peak point Laplacian (ppLapl) calculated at the position in the stone with maxHU. Two prediction models were previously published methods (ppLapl-maxHU and maxHU) and the third was derived from the previous results based on the k-nearest neighbors (kNN) algorithm (kNN-ppLapl-maxHU). The three methods were evaluated on this new independent stone dataset. The reference standard was the CT vendor’s DE-CT application for kidney stones.

**Results:**

Altogether 124 participants (59 ± 14 years, 91 men) with 106 non-UA and 37 UA stones were evaluated. For classification of UA and non-UA stones, the sensitivity, specificity, and accuracy were 100% (37/37), 97% (103/106), and 98% (140/143), respectively, for kNN-ppLapl-maxHU; 95% (35/37), 98% (104/106), and 97% (139/143) for ppLapl-maxHU; and 92% (34/37), 94% (100/106), and 94% (134/143) for maxHU.

**Conclusion:**

A quantitative SE-CT method (kNN-ppLapl-maxHU) can classify UA stones with accuracy comparable to DE-CT.

**Key Points:**

• *Single-energy CT is the first-line diagnostic tool for suspected renal colic.*

• *A single-energy CT method based on the internal urinary stone attenuation distribution can classify urinary stones into uric acid and non-uric acid stones with high accuracy.*

• *This immensely increases the availability of in vivo stone analysis.*

## Introduction

Urinary stone disease continues to be an increasing reason for health care admissions worldwide, with an incidence of 7% among women and 11% among men in the USA in 2010 [1]. In northern Europe, an increasing frequency of uric acid (UA) stones has been reported [2]. Approximately 7–11% of all urinary stones are UA stones [3].

Distinguishing UA from non-UA stones is of particular interest for the urologist, as the former can be treated with alkalization of the urine, allowing for secondary prophylaxis after ex vivo composition analysis of a passed or surgically removed stone [4–7]. Ideally, a UA stone is identified and dissolved in vivo, to facilitate stone passage and obviate surgical removal [8, 9]. At present, in vivo stone composition analysis is mostly accomplished with dual-energy CT (DE-CT), which has shown high accuracy in several studies [10–15].

Non-enhanced single-energy CT (SE-CT) is the first-line diagnostic tool for suspected renal colic and is able to detect nearly all urinary stones with high specificity. It is highly reproducible for measuring stone size and useful for predicting spontaneous stone passage [6, 14, 16]. If, in addition, this method could also predict the composition of the stone, the patient would be able to leave the emergency room with a tailored treatment on the first day of radiologic diagnosis [9].

In a recent exploratory study on 126 urinary (22 UA and 104 non-UA) stones, Lidén correlated quantitative CT parameters in the stones to the chemical composition [17]. The highest (peak) attenuation (maxHU) of a single voxel in the stone showed to be a powerful predictor of stone composition, but, to increase the specificity, Lidén proposed a purely quantitative SE-CT method called peak point Laplacian/maxHU (ppLapl-maxHU). The ppLapl-maxHU method uses the peak attenuation in the examined stone, measured in Hounsfield units (HUs), together with the difference between this peak attenuation and the weighted mean attenuation of the surrounding voxels (Laplacian), to obtain a cutoff value of 195 HU/1000 HU (ppLapl/maxHU) for differentiating UA from non-UA stones. The cutoff values of the ppLapl-maxHU method resulted in high sensitivity and specificity (95% and 99% respectively), but with the major limitation that the cutoff values were defined post hoc, possibly causing an overestimation of the accuracy in the study. External validation on a separate dataset is therefore needed. An important advantage of the measures maxHU and ppLapl, compared to the mean attenuation often used for attenuation measurements of urinary stones, is that these two measures are point estimates, and therefore independent of segmentation parameters and reproducible.

Additional analysis of the UA- and non-UA stone data in the previous study suggested that machine learning, avoiding the sharp cutoff lines in the ppLapl-maxHU method, might perform even better than the original method [17]. K-nearest neighbors (kNN) is a simple machine learning algorithm that uses the features of annotated cases and, through a majority vote, classifies new data according to how their neighbors in a multi-dimensional space were classified [18].

Using the same data as used in the recently developed ppLapl-maxHU method [17], a kNN algorithm was derived (kNN-ppLapl-maxHU). We hypothesized that the kNN-ppLapl-maxHU and the ppLapl-maxHU methods could differentiate UA from non-UA stones on previously unseen SE-CT with a sensitivity and specificity comparable to DE-CT and greater than using only the peak attenuation (maxHU) of the stones.

The purpose of the present study was to prospectively validate two previously published (ppLapl-maxHU and maxHU), and one derived (kNN-ppLapl-maxHU) quantitative single-energy CT methods for classifying uric acid stones on a separate, previously unseen stone dataset.

## Materials and methods

This study was approved by the Regional Research Ethics Board. Written informed consent was obtained from all prospectively and retrospectively included participants.

### Study participants

**Fig. 1:**
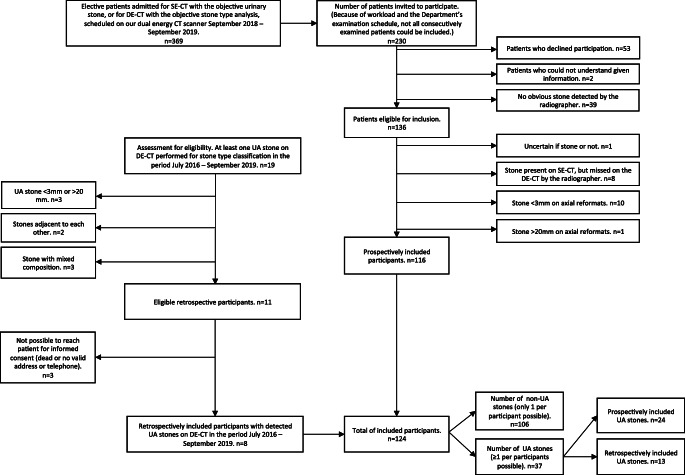
Flowchart of inclusion. SE-CT, single-energy CT; DE-CT, dual-energy CT; UA, uric acid; non-UA, non uric-acid

Between September 2018 and September 2019, 116 study participants planned for elective urinary stone CT examination on our DE-CT scanner, with at least one 3–20-mm urinary stone, were prospectively included in the study to create a test dataset for validating the index tests described below (65% of prospective, eligible participants: due to fluctuating workload, not all potentially eligible patients were asked to participate). Because of the known low prevalence of UA stones in our patient population, all eligible UA stones were included, but to keep the heterogeneity of the non-UA stones as high as possible, only one non-UA stone per participant. For inclusion and exclusion criteria, see Fig. 1.

A preliminary reading of the CT scans was performed in patients accepting participation. Once the radiographer had established that there was at least one urinary stone fulfilling the criteria, the patient was included and a reference DE-CT was performed. The definite decision about eligibility for inclusion was later made by a radiologist with 15 years’ experience in reading abdominal CTs (J.J.).

Based on the expected low number of UA stones in our patient population, we made a pre-study-planned, additional retrospective inclusion of UA stones demonstrated on DE-CT between August 2016 and August 2018, where a SE-CT scan was available. One DE-CT stone analysis examination during the prospective inclusion period was missed and therefore retrospectively included. In total, eight (73%) of the retrospectively eligible participants were included.

The sample size of ≥ 35 UA and 100–150 non-UA stones was estimated with the objective of reaching the lower limit of a 95% confidence interval (CI) of 80%, for the hypothesized sensitivity of 95%, and the lower limit of 95% for the hypothesized specificity of 97%.

### Study protocol and technical specifications

All study participants were examined on a 2 × 128 channel dual-source system (Somatom Definition Flash, Siemens) using our local routine single-energy and a dual-energy protocol, for scan parameters, see Table 1. The mean dose length product was 185 ± 55 mGy*cm (range 98–432) and the mean volume CT dose index was 4.5 mGy (range 2.7–10) for the SE-CT and 189 ± 101 mGy*cm (range 54–637) and 16 mGy (range 5.6–41) for the DE-CT examinations, respectively. The mean DE-CT scan length was 10.7 (range 3.8–61) cm.

**Table 1:** Scan parameters

Scan parameter	Single-energy CT (SE-CT)	Dual-energy CT (DE-CT)
Acquisition	128 × 0.6	32 × 0.6
Filter	SAFIRE I30f3	D30f
kVp	120	100/Sn 140
Quality reference mAs	70	210/162
Pitch	1.2	0.7
Rotation time	0.5	0.5
CARE-kV	Off	N/A
CARE dose 4D	On	On
Scan area	Upper kidney poles to pelvic floor	Limited, surrounding the stone(s)
Field of view (mm)	420	300
Slice thickness/increment (axial reformat) (mm)	1/1	2/1
Slice thickness/increment (coronal/sagittal reformat)	3/3	–

*Sn*, tin pre-filtration; *kV*, kilovolt; *kVp*, kilovoltage peak

The SE-CT and DE-CT reformats were pseudonymized and saved with different keys in separate folders in the local picture archiving and communication system (PACS, Sectra).

### Index tests

The three index tests were created using the same data from a previous study consisting of 126 stones with known pure UA/non-UA composition [17]. While the ppLapl-maxHU and the maxHU methods were published in the previous study, the kNN-ppLapl-maxHU method was derived for the current study. No data from the included stones in the current study was used for the development of the kNN method. None of the three index tests is commercially available at present.

#### Index test—ppLapl-maxHU

**Fig. 2:**
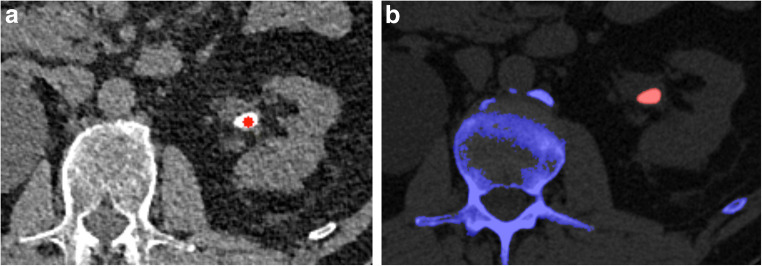
Index test (**a**) and reference test (**b**) evaluating a left-sided 11 × 5 mm uric acid (UA) kidney stone in a 66-year-old man. **a** Index test: 1-mm single-energy non-enhanced axial CT scan after export to external software and marking of the kidney stone by the radiologist. The red star indicates UA composition according to the peak point Laplacian-maximum attenuation (ppLapl-maxHU) algorithm: in this case, the combination of a peak point attenuation of 693 HU (< 1000 HU) and a difference between this peak point attenuation and the mean of the surrounding 26 voxels of 98 HU (< 195 HU). **b** Reference test: Post-processed axial dual-energy CT image using the vendor’s stone composition analysis application. Red indicates stone with UA composition

**Fig. 3:**
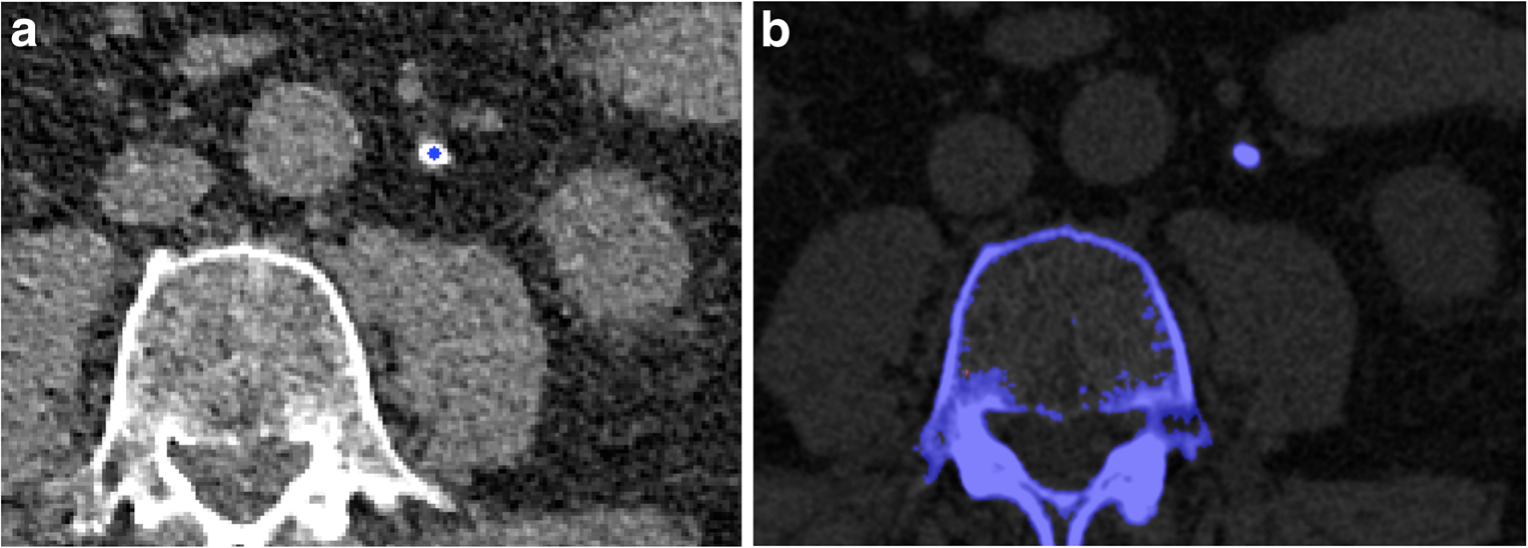
Index test (**a**) and reference test (**b**) evaluating a left-sided, 5 × 3 mm non-uric acid (non-UA) ureteral stone in a 78-year-old man. **a** Index test: 1-mm single-energy, non-enhanced axial CT scan after export to external software and marking of the ureteral stone by the radiologist. The blue star indicates non-UA composition, according to the peak point Laplacian-maximum attenuation (ppLapl-maxHU) algorithm: A peak point attenuation of ≥ 1000 HU (in this case 1398 HU) *or* a difference between this peak point attenuation and the weighted mean of the surrounding voxels of ≥ 195 HU (in this case, 182 HU) defines this as a stone of non-UA composition. **b** Reference test: Post processed axial, dual-energy CT image using the vendor’s stone composition analysis application. The blue color indicates stone with non-UA composition

**Fig. 4:**
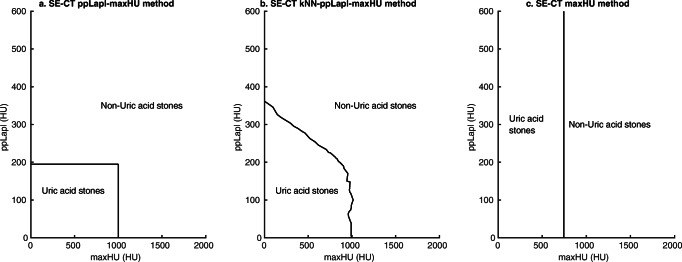
Single-energy CT (SE-CT) methods for classification of uric acid (UA) stones based on maximal attenuation (maxHU) and peak point Laplacian (ppLapl). The three different SE-CT methods for the classification of UA and non-UA stones differ in the cutoffs for the radiomics maxHU (the highest attenuating voxel in the stone) and ppLapl. **a** Rigid cutoffs according to the original method [14]; **b** nearest neighbor method; and **c** maxHU method

The two quantitative variables used for stone type prediction were (1) the highest attenuating voxel in the stone (maxHU) and (2) the value at the same position as for maxHU in a scaled Laplacian filtered image (ppLapl). An interpretation of the ppLapl is a computation of the attenuation difference between the highest attenuating voxel value and the weighted mean of the surrounding 26 voxels [17]. The pseudonymized SE-CT images were exported to MATLAB R2019a (MathWorks Inc.), where, 4 weeks after completing the inclusion process, a radiologist (J.J.) marked each included stone using previously developed, semi-automatic MATLAB software to generate the maxHU and ppLapl-maxHU values (Figs. 2a and 3a). The application assigned a red color dot for UA stones and a blue dot for non-UA stones, using the cutoff values proposed in a previous study [17] (Figs. 2 and 4a).

#### Index test—kNN-ppLapl-maxHU

The kNN machine learning prediction method is based on the ppLapl-maxHU method. The prediction model was created in MATLAB using the nine nearest neighbors, a standard setting, and Euclidean distance. The method thereby uses the maxHU and ppLapl values of all the known stones from the previously annotated dataset, compares them with the position of an unknown stone in a scatter plot, and performs a “majority vote” between the nine stones closest to the unknown stone. With only two variables in the kNN, the model can be illustrated as a curved line separating the predicted UA from non-UA stones in a scatter plot (Fig. 4b). The difference compared to the rectangular area of UA stones in the original method (Fig. 4a) is consequently the smoother curved line separating the UA from the non-UA stones.

#### Index test—maxHU

The third index test used only the optimal maxHU, 745 HU, defined previously [17] (Fig. 4c).

### Reference test/ground truth

The stone classification (UA vs. non-UA) in DE-CT was used as ground truth for each included stone, as DE-CT can reliably differentiate pure UA stones from non-UA stones [10–13]. The DE-CT images were analyzed, using the kidney stone application in Syngo.Via (Siemens), by a radiologist (J.J.) in conjunction with the inclusion procedure. If the stone was color-coded > 80% red (visual approximation), it was considered a UA stone, and if > 80% blue, a non-UA stone (see Figs. 2b and 3b). The remaining stones (*n* = 7) were considered UA/non-UA mixed stones and removed from further analysis, since the DE-CT has lower reliability for mixed stones and consequently is insufficient as a reference test [19–22].

The largest diameter of each stone was measured manually in the axial plane 3-mm slice using the caliper tool in the PACS workstation with a soft window setting of C50/W400 and a zoom level of pixel-to-pixel × 8.

### Statistical analysis

Analyses were performed using IBM SPSS for Mac OS, v26.0.0.0 (SPSS Inc.), and MATLAB.

Sensitivity and specificity for, and accuracy of the prediction of UA stones, using binomial distribution and 95% CIs were calculated for the SE-CT classification methods (kNN-ppLapl-maxHU, ppLapl-maxHU, and maxHU).

This analysis was made primarily for all stones grouped together. Secondary analyses were performed: (1) prospectively included stones and (2) including only one UA (the most caudally positioned) or non-UA stone per patient.

Statistical significance of the difference between the index methods was tested using McNemar’s test (level of significance: *p* < 0.05).

## Results

Altogether 124 participants (59 ± 14 years, 91 men) with 106 non-UA and 37 UA stones were evaluated. Thirteen men had UA stones (14%) and 78 had non-UA stones (86%). Five women (15%) had UA stones, whereas 28 (85%) had non-UA stones. None of these stones has been used for the development of the index methods. Table 2 shows the stone characteristics. The mean age in the UA group was 69 ± 8 years (range 50–77) and in the non-UA group 58 ± 14 (21–89).

**Table 2:** Stone characteristics

	All (*n* = 143)	Non-UA (*n* = 106)	UA (*n* = 37)	UA misclassified as non-UA by ppLapl-maxHU (*n* = 2)**	Non-UA misclassified as UA by ppLapl-maxHU (*n* = 2)**	Non-UA misclassified as UA by kNN-ppLapl-maxHU (*n* = 3)**	UA misclassified as UA by kNN-ppLapl-maxHU (*n* = 0)
Max HU (HU)*	1092 ± 421 (223–1796)	1286 ± 292 (536–1796)	537 ± 155 (223–794)	602 (450–754)	873 (826–919)	760 (536– 919)	-
ppLapl (HU)*	240 ± 110 (47–546)	282 ± 96 (51–546)	122 ± 39 (47–212)	206 (201–212)	95 (79–110)	133 (79–209)	-
Size (mm)*	6.5 ± 3.0 (3.0–19)	6.3 ± 2.5 (3.0–15)	7.3 ± 4.1 (3.0–19)	4.0 (3.0–4.9)	9.4 ± 0.8 (8.8–10)	8.3 (6.1–10)	-

*SD*, standard deviation; *UA*, uric acid; *Max HU*, maximum attenuation in a single voxel in a stone; *HU*, Hounsfield units; *ppLapl-maxHU-method*, peak point Laplacian-maximum attenuation, SE-CT method using the highest attenuation voxel in the stone and the weighted mean of the surrounding voxels to classify a urinary stone into UA or non-UA; *kNN-ppLapl-maxHU-method*, machine learning modification of the ppLapl-maxHU method using the nine nearest neighbors for classification

*Note*: *Values are mean ± standard deviation (range). ** Standard deviation not calculated because of low number of stones

**Fig. 5:**
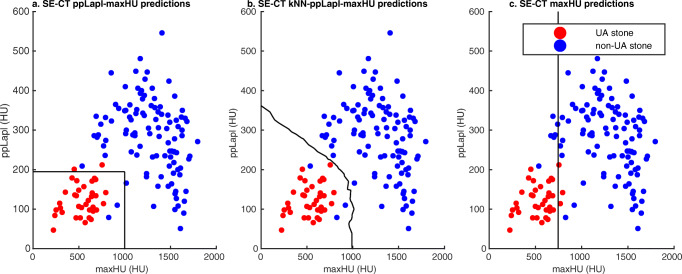
Scatter plots of the three different single-energy CT (SE-CT) methods for classification of uric acid (UA) and non-UA stones. Red dots: UA stones defined by dual-energy CT (DE-CT). Blue dots: Non-UA stones defined by DE-CT. **a** ppLapl-maxHU method. **b** kNN-ppLapl-maxHU method. **c** maxHU method

Cross-tabulations of the results of the kNN-ppLapl-maxHU, ppLapl-maxHU, and maxHU methods by the results of the reference DE-CT are shown in Table 3. Scatter plots with the distribution of maxHU and ppLapl for all stones, with cutoffs according to the three different SE-CT classification methods are shown in Fig. 5. Table 4 shows sensitivity, specificity, and accuracy for the three methods. Table 5 displays sensitivity, specificity, and accuracy of subgroups (a) prospectively included stones and (b) only one included UA stone per patient.

**Table 3:** Cross-tabulations of single-energy CT (SE-CT) classification of urinary stones into uric acid (UA) and non-UA stones using dual-energy CT (DE-CT) as reference. (a) kNN-peak point Laplacian-maxHU (kNN-ppLapl-maxHU); (b) peak point Laplacian-maxHU (ppLapl-maxHU); (c) maxHU

		Dual-energy CT		
		Non-UA	UA	Total
a. kNN-ppLapl-maxHU * Dual-energy CT				
kNN-ppLapl-maxHU	Non-UA	103	0	103
	UA	3	37	40
	Total	106	37	143
*Sensitivity: 100% (95%CI 91–100%)*				
*Specificity: 97% (95%CI 92–99%)*				
*Accuracy: 98% (95%CI 94–100%)*				
b. ppLapl-maxHU * Dual-energy CT				
ppLapl-maxHU	Non-UA	104	2	106
	UA	2	35	37
	Total	106	37	143
*Sensitivity: 95% (95%CI 82–99%)*				
*Specificity: 98% (95%CI 93–100%)*				
*Accuracy: 97% (95%CI 93–99%)*				
c. maxHU * Dual-energy CT				
maxHU	Non-UA	100	3	103
	UA	6	34	40
	Total	106	37	143
*Sensitivity: 92% (95%CI 78–98%)*				
*Specificity: 94% (95%CI 88–98%)*				
*Accuracy: 94% (95%CI 88–97%)*				

Sensitivity and specificity for the prediction of UA stones

*ppLapl-maxHU*, peak point Laplacian-maximum attenuation, SE-CT method using the highest attenuation voxel in the stone and the weighted mean of the surrounding voxels to classify a urinary stone into UA or non-UA; *kNN-ppLapl-maxHU*, machine learning modification of the ppLapl-maxHU method using the nine nearest neighbors for classification; *maxHU*, SE-CT method using the single voxel with the highest attenuation in a stone as cutoff (< 745 ➔ UA stone)

Dual-energy CT: Kidney stone application Syngo.Via

**Table 4:** Sensitivity, specificity, and accuracy for the prediction of UA stones by three single-energy CT methods. Dual-energy CT as a reference standard

		Sensitivity, 95% CI	Specificity, 95% CI	Accuracy, 95% CI
All included stones (37 UA, 106 non-UA).	kNN-ppLapl-maxHU	100% (37/37) 91–100%	97% (103/106) 92–99%	98% (140/143) 94–100%
	ppLapl-maxHU	95% (35/37) 82–99%	98% (104/106) 93–100%	97% (139/143) 93–99%
	maxHU	92% (34/37) 78–98%	94% (100/106) 88–98%	94% (134/143) 88–97%

*UA*, uric acid; *Non-UA*, non-uric acid; *95% CI*, 95% confidence intervals; *ppLapl-maxHU*, peak point Laplacian-maximum attenuation, single-energy CT method using the highest attenuation voxel in the stone and the weighted mean of the surrounding voxels to classify a urinary stone into UA or non-UA; *kNN-ppLapl-maxHU*, machine learning modification of the ppLapl-maxHU method using the nine nearest neighbors for classification; *maxHU*, single-energy CT method using the single voxel with the highest attenuation in a stone as cutoff (< 745 ➔ UA stone)

**Table 5:** Sensitivity, specificity, and accuracy for the prediction of UA-stones by three single-energy CT methods (a) prospectively included stones, (b) only one included UA-stone per patient. Dual-energy CT as a reference standard

		Sensitivity, 95% CI	Specificity, 95% CI	Accuracy, 95% CI
a. Prospectively included stones (24 UA, 106 non-UA).	kNN-ppLapl-maxHU	100% (24/24) 86–100%	97% (103/106) 92–99%	98% (127/130) 93–100%
	ppLapl-maxHU	92% (22/24) 73–99%	98% (104/106) 93–100%	97% (126/130) 92–99%
	maxHU	88% (21/24) 68–97%	94% (100/106) 88–98%	93% (121/130) 87–97%
b. Only one included UA-stone per patient (18 UA, 106 non-UA).	kNN-ppLapl-maxHU	100% (18/18) 82–100%	97% (103/106) 92–99%	98% (121/124) 93–100%
	ppLapl-maxHU	94% (17/18) 73–100%	98% (104/106) 93–100%	98% (121/124) 93–100%
	maxHU	89% (16/18) 65–99%	94% (100/106) 88–98%	94% (116/124) 88–97%

UA, uric acid; Non-UA, non-uric acid; 95% CI = 95% confidence intervals; ppLapl-maxHU = peak point Laplacian-maximum attenuation, single-energy CT method using the highest attenuation voxel in the stone and the weighted mean of the surrounding voxels to classify a urinary stone into UA or non-UA; *kNN-ppLapl-maxHU*, machine learning modification of the ppLapl-maxHU method using the nine nearest neighbors for classification; *maxHU*, single-energy CT method using the single voxel with the highest attenuation in a stone as cutoff (< 745 ➔ UA stone)

The area under the receiver operating characteristics (ROC) curve (AUC) for the kNN-ppLapl-maxHU, as well as the maxHU, was 0.99. The AUC cannot be computed for the ppLapl-maxHU method with two static cutoffs. Although not statistically significant (*p* = 0.06) according to the McNemar test, there was a tendency towards higher accuracy for the kNN-ppLapl-maxHU method (98%) compared to the maxHU method (94%). The 95% CI for the difference was - 0.5 to 9.5 percentage points.

## Discussion

Single-energy CT is the first-line modality for the detection of urinary stones, whereas the in vivo stone analysis is usually conducted with dual-energy CT, with limited availability in most emergency radiology settings [23]. The purpose of this study was to prospectively validate two previously published and one derived quantitative method (kNN-ppLapl-maxHU) for in vivo prediction of uric acid (UA) stone type using the first-line single-energy CT scan. The kNN-ppLapl-maxHU method obtained a sensitivity for UA stones, 3–20 mm, of 100% (37/37), a specificity of 97% (103/106), and an accuracy of 98% (140/143). The accuracy of both the ppLapl-maxHU and the maxHU method was also high, 97% and 94%, respectively.

The close correlation between the attenuation and composition of a urinary stone is well known, and also the considerable overlap between UA and non-UA stones [24–26]. The kNN-ppLapl-maxHU method is purely quantitative and combines the highest attenuation within a stone with the attenuations of the surrounding 26 voxels, giving an estimate of the stone attenuation peakedness. The radiomics used in the kNN-ppLapl-maxHU method have a logical interpretation. A small, calcium-based stone can have the same peak attenuation as a larger, UA-based stone, but the difference in attenuation between the highest attenuating voxel and the surrounding voxels is generally larger in the calcium than in the UA stone [17]. This corresponds well with previously published retrospective non-validated results. Nakada et al [24] used stone size and attenuation to analyze a sample of 17 UA and 82 non-UA stones and reached an accuracy of 86% (85/99); Ganesan et al [27], using stone size, attenuation, and attenuation distribution in a retrospective cohort of 52 calcium oxalate and 48 UA stones, reached an accuracy of 90%; and Zhang et al [28] used stone texture analysis in a sample of 18 UA and 32 non-UA stones and reached a sensitivity and specificity of 94%.

The original methods that are prospectively validated in the present study (ppLapl-maxHU and maxHU) were developed by Lidén [17] using the distribution of the clusters of UA and non-UA stones in a scatter plot showing maxHU and ppLapl. Also, the kNN-ppLapl-maxHU algorithm in the present study was derived from only the previous data before analyzing the new data that was collected for the present study.

Consequently, a main strength of the present study compared to the previous is that the three tested algorithms were predefined, thereby avoiding the problem of overfitting. Although thirteen UA stones were retrospectively included, the results were similar when using only the prospectively included stones.

The kNN-ppLapl-maxHU model uses a previously acquired dataset [17] to classify a stone of unknown type through a “majority vote” between its nine closest neighbors in a scatter plot. Compared to the original method’s rigid cutoff of 195 HU/1000 HU, the proposed kNN method is appealing, as it creates a smoother curved line between the UA and non-UA stones. In our material, however, both methods had excellent accuracy (97–98%) and either method can be used. Given this high accuracy for both methods, achieving a statistically significant difference in accuracy between them would demand a very high number of included stones, which is difficult to achieve considering the relatively low prevalence of UA stones. An advantage of the kNN-ppLapl-maxHU method is its plasticity; a future addition of more training examples to the algorithm is likely to further increase its accuracy. Although our hypothesis that the accuracy of the kNN-ppLapl-maxHU would be higher than the accuracy of the method using only maxHU could not be statistically proven, there was a strong tendency towards a higher accuracy (98% vs 94% (*p* = 0.06)).

To our knowledge, this is the first prospective validation study of a SE-CT method for in vivo classification of UA and non-UA urinary stones. The results of this study are comparable to previously published results for DE-CT [11–13].

This study has limitations. Three stones in the retrospective and four in the prospective subgroup were of mixed composition (visually 20–80% UA in the DE-CT application) and were removed from the analysis. Although a recent phantom study showed promising results in demonstrating the main stone component, using a machine learning algorithm on spectral detector DE-CT [29], no previous study has shown that DE-CT can reliably classify mixed stones in vivo. A valid reference standard was consequently not available. Most studies on DE-CT classification of UA vs. non-UA stones have been performed on pure or nearly pure (80–90%) stones [13, 19, 21, 30–33]. Consequently, the index tests are not designed for classification beyond UA/non-UA stones.

Thirteen UA stones in eight patients were included retrospectively to achieve a sufficient number of UA stones. The index tests are purely quantitative and the radiologist performing them was blinded to the results of the reference standard. The risk for bias by retrospectively including eight participants with confirmed UA stone was considered low. In addition, the sensitivity and specificity were virtually unchanged when only the prospectively included stones were analyzed; merely the CIs became broader. Because of the known low prevalence of UA stones in our population, all UA stones in a patient meeting the inclusion criteria were included, similar to previous studies [10, 12, 13, 20, 28, 33–36] which may lower the heterogeneity in the UA group. In non-UA stones, the sample size could be reached with independent stones from different patients. The different inclusion strategies may thereby introduce a lower heterogeneity between UA stones, but the risk of introducing a systemic bias is considered low. When only the UA stone with the most caudal position per patient was analyzed, the sensitivity and specificity remained virtually unchanged, but with broader CIs.

The validation is made for the current settings in the CT scanner family used in this study. Validation tests on scanners from various CT providers need to be performed before the method can be generalized to other CT manufacturers [37]. This is an important limitation, but there is good reason to believe that similar results can be achieved after optimization, regardless of CT manufacturer, since the ppLapl-maxHU radiomics are based on the physical properties of the different stone types. Furthermore, the need for optimization of the present method depending on CT manufacturer is no different from the need for optimization depending on scanner type, of other methods, for example, a DE-CT stone type classification method.

In conclusion, this study demonstrates that a purely quantitative single-energy CT method can classify uric acid (UA) and non-UA stones in a previously unseen dataset, with accuracy comparable to dual-energy CT, enabling immediate stone classification when a urinary stone is detected. Considering the lower cost, better availability, and lower radiation exposition, this is a promising alternative to dual-energy CT for in vivo characterization of urinary stones.

## Abbreviations

AUC: Area under the receiver operating characteristics curve
CI: Confidence interval
DE-CT: Dual-energy computed tomography
HU: Hounsfield unit
kNN: K-nearest neighbors
maxHU: Maximal attenuation in a region of interest
non-UA: Non-uric acid
PACS: Picture archiving and communication system
ppLapl: Peak point Laplacian
ROC: Receiver operating characteristics
SE-CT: Single-energy computed tomography
UA: Uric acid
